# Cephalexin-Induced Leukocytoclastic Vasculitis

**DOI:** 10.7759/cureus.26545

**Published:** 2022-07-04

**Authors:** Wesley Tang, Jennie Tan

**Affiliations:** 1 Internal Medicine, Kettering Medical Center, Dayton, USA; 2 Internal Medicine, Kettering Medical Center, Kettering, USA

**Keywords:** antibiotics, leukocytoclastic vasculitis, hypersensitivity vasculitis, cephalosporins, cephalexin

## Abstract

Although allergies to antibiotics are commonly stated, allergies to cephalosporin antibiotics are uncommonly reported. Furthermore, dermatologic and systemic reactions from cephalosporin exposure involving end-organ damage are rare. We present a case of cephalexin, a cephalosporin, induced hypersensitivity vasculitis causing characteristic non-blanching purpuric lesions along with acute kidney failure necessitating hospitalization, corticosteroids, and initiation of hemodialysis.

## Introduction

Reactions to antibiotics are common, with roughly 2.2% of all hospitalized patients developing a cutaneous drug reaction [1]. The majority of these reactions are caused by trimethoprim-sulfamethoxazole, penicillin, amoxicillin, and ampicillin [2]. It is estimated that the prevalence of self-reported beta-lactam allergy is up to 10% of hospitalized patients [3]. The incidence of reactions to cephalosporins is not known. However, reactions to cephalosporins are estimated to be magnitudes lower than penicillin with an estimated risk range between 0.0001% and 0.1% for each treatment course [4]. Dermatologic and systemic illnesses with end-organ failure are more uncommon manifestations. Here, we present a case report of leukocytoclastic vasculitis caused by cephalexin, a cephalosporin. Leukocytoclastic vasculitis is a cutaneous small-vessel vasculitis characterized by cutaneous manifestations in the form of palpable purpura, and rarely ulcerations, vesicles, and bullae [5]. We present a case of a patient that was prescribed cephalexin for a urinary tract infection, and developed hypersensitivity vasculitis, leading to end-stage renal disease necessitating the initiation of hemodialysis.

## Case presentation

**Figure 1:**
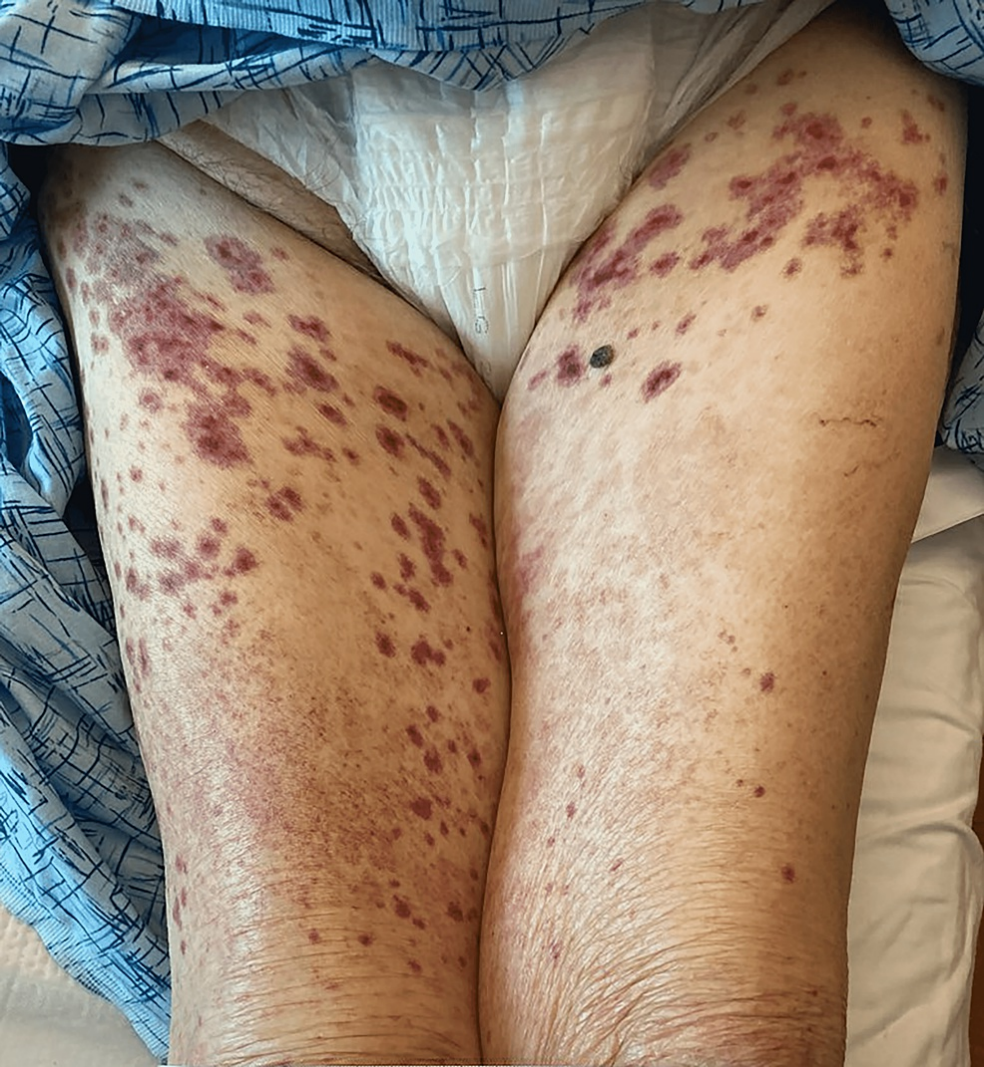
Non-blanching rash of lower extremities consistent with hypersensitivity vasculitis

**Figure 2:**
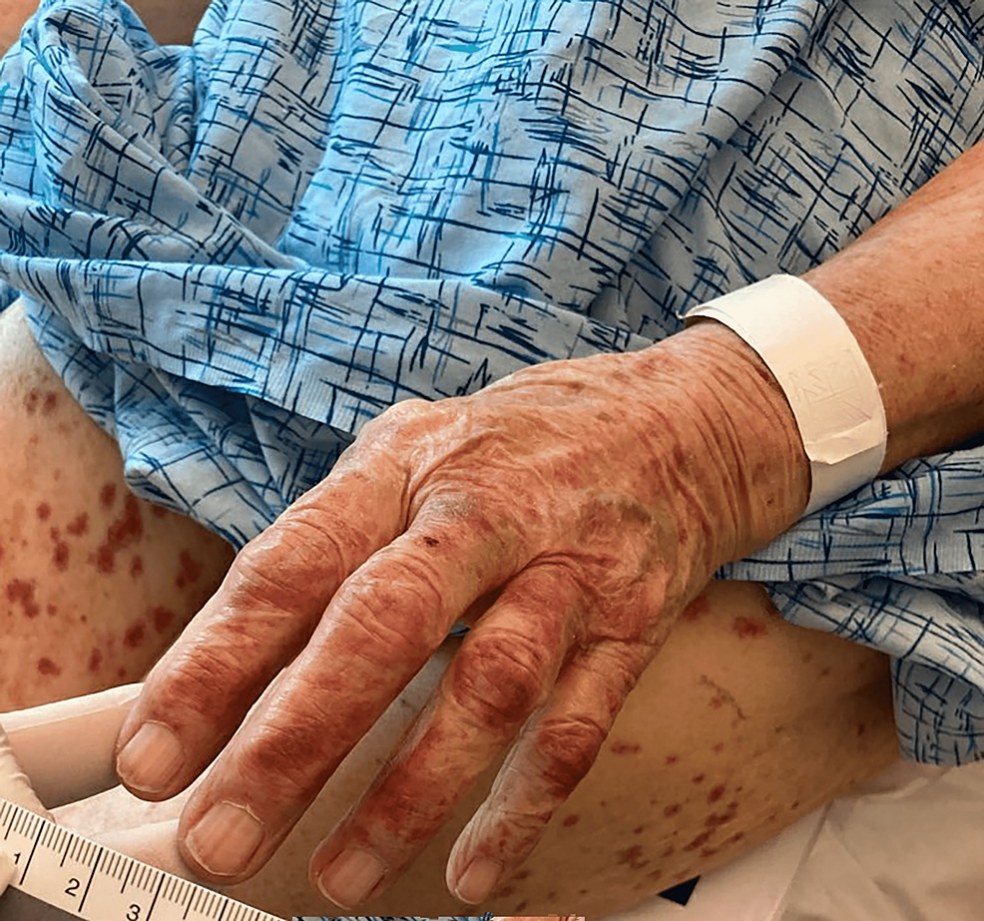
Non-blanching rash of upper extremity

The patient was a 91-year-old female with a past medical history of chronic kidney disease stage IV, diet-controlled diabetes mellitus type 2, and hypothyroidism on replacement therapy, who presented to an outpatient clinic for right calf and foot cellulitis of one-month duration. She was treated with cephalexin (500 mg by mouth three times a day for seven days), and seven days after completion of antibiotic therapy, she developed a bilateral lower extremity and abdominal rash, along with arthralgias and generalized weakness. She denied any chest pain, headache, signs of overt bleeding, or significant weight loss. The patient further denied any known drug allergies or reactions to medications. She presented to the hospital two weeks after initiation of cephalexin. Upon initial evaluation, she was hypertensive with a blood pressure of 183/69. She was afebrile. On her abdomen, hands, and legs, she had a diffuse palpable purpuric rash that was non-tender to palpation (Figures 1-2).

Her skin lesions were non-blanching. Her right lower extremity was dry and scaly without erythema or swelling. Laboratory workup was significant for C-reactive protein (CRP) 118 mg/L, erythrocyte sedimentation rate (ESR) 14 mm/hr, creatinine of 4.2 mg/dL (baseline creatinine 2.7 mg/dL), estimated glomerular filtration rate of 8 mL/min/1.73m2, serum potassium of 5.7 mmol/L, and serum bicarbonate of 14 mmol/L. Complete blood count (CBC) was significant for hemoglobin at 11.2 g/dL and platelet count at 205 K/uL. Urinalysis revealed +3 blood, 200 mg/dL protein, and trace bacteria. Chest X-ray was performed and interpreted as non-acute.

Infectious disease was consulted and determined that there was no indication for topical or systemic antibiotics as the patient did not have a fever or leukocytosis, and that her right lower extremity rash was inconsistent with cellulitis. Given her rash, acute kidney injury, and her history of antibiotic use, her presentation was consistent with hypersensitivity vasculitis. Initially, she was given intravenous fluid boluses and started on intravenous methylprednisolone and sodium bicarbonate infusion. Renal imaging revealed her chronic renal disease and a simple right renal cyst. Further work up for kidney injury included creatine kinase, uric acid, and lactate dehydrogenase which were within normal limits. Her blood cultures showed no growth. She was not given any topical or systemic antibiotics. She also complained of mid-back pain, and imaging revealed a T4 compression fracture, managed conservatively after neurosurgical evaluation. After admission, she had developed right upper extremity swelling. Ultrasound imaging revealed a superficial vein thrombosis in her right cephalic vein, as well as a large hematoma (9.8 x 3.1 x 6.0 cm) in the right biceps brachii. An MRI did not show a biceps brachii tendon rupture. Symptoms improved with conservative management.

After nine days of hospitalization, she began developing signs of hypervolemia with pulmonary edema, extremity edema, and shortness of breath from hypoxic respiratory failure requiring 4 liters of oxygen via nasal cannula. A hemodialysis catheter was placed, and she underwent hemodialysis for three consecutive days with an improvement of her symptoms. Her hemoglobin dropped to 6.5 g/dL, which was likely multifactorial due to her acute illness, chronic renal disease, and she required two units of packed red cells.

Her rash continued to improve, and she was transitioned to prednisone 20 mg daily after two days. However, her creatinine remained elevated at about 4.0 mg/dL, and her urine output remained poor. At the time of discharge, as there was no renal improvement and her rash had resolved, her prednisone was discontinued. She was scheduled to continue regular outpatient hemodialysis. In 12 months of follow-up, the patient continues with hemodialysis, without recurrence of rash, arthralgias, or weakness.

## Discussion

Leukocytoclastic vasculitis is a condition affecting the small vessels of the skin that can involve internal organs. In a study of 239 patients with leukocytoclastic vasculitis and visceral involvement, 1/3 of patients had gastrointestinal or renal involvement [6]. Drug-associated cutaneous vasculitis is due to immunocomplex deposition/type III hypersensitivity reaction. The immune complexes with antigen formed circulate until deposition into blood vessel walls. Activated neutrophils release proteolytic enzymes, and endothelial cell membrane integrity is compromised [7]. 

Most cases of leukocytoclastic vasculitis are idiopathic in nature, though often attributed to drugs and infections when known [8]. The diagnosis of leukocytoclastic vasculitis is primarily a clinical diagnosis [9]. Skin lesions are characterized as multiple non-blanching purpuric lesions, often limited to the lower extremities. Other skin findings can include bullae, papules, plaques, nodules, ulcers, and livedo reticularis. If a skin biopsy of a purpuric lesion is to be performed, the typical histologic findings include perivascular neutrophil degeneration, extravasation of erythrocytes, and necrosis of the vessels [10].

We performed a PubMed search from 1950 to the present day to find a link between cephalosporin exposure and hypersensitivity vasculitis. We used the search terms ‘cephalosporin’, ‘cephalexin’, ‘hypersensitivity vasculitis’, ‘leukocytoclastic vasculitis’, and found a total of three case reports linking a cephalosporin with dermatologic manifestations. One case report implicated ceftriaxone, a second implicated cefazolin, and a third implicated cefuroxime [5,11,12].

In our case, the patient presented to the emergency department complaining of bilateral lower extremity and abdominal rash after taking cephalexin two weeks prior. Initially, she was evaluated for systemic infections and investigations were largely unremarkable. Instead, the patient was prescribed corticosteroids, given the evidence of acute on chronic kidney injury with gradual improvement. Unfortunately, given her pre-existing condition of chronic kidney disease stage IV, this patient would ultimately be discharged from the hospital requiring ongoing hemodialysis.

## Conclusions

When a case of leukocytoclastic vasculitis emerges, the first step should be a thorough review of all medications and stopping offending medications when feasibly possible. In the absence of systemic signs, discontinuation of the offending agent and close monitoring is usually sufficient. Corticosteroids are often offered if there is evidence of end-organ damage. More severe cases may warrant further immunosuppressive agents such as azathioprine, mycophenolate mofetil, methotrexate, or rituximab.
